# Neutrophil-to-lymphocyte ratio as a prognostic biomarker in lung cancer patients treated with PD-1/PD-L1 inhibitors: A systematic review and meta-analysis

**DOI:** 10.17305/bb.2026.14145

**Published:** 2026-05-28

**Authors:** Yong Zeng, Junlin Gu, Weiqiong Chen, Wensheng He, Jianjun Luo

**Affiliations:** 1 Department of Pharmacy, The Affiliated Dazu’s Hospital of Chongqing Medical University, Chongqing, China

**Keywords:** Lung cancer, neutrophil-to-lymphocyte ratio, PD-1, PD-L1, prognosis

## Abstract

Lung cancer (LC) continues to be a leading cause of cancer-related mortality, highlighting the need for reliable and accessible biomarkers to identify patients at higher risk of poor outcomes during immunotherapy. This systematic review and meta-analysis aimed to evaluate the prognostic value of the neutrophil-to-lymphocyte ratio (NLR) in patients with LC receiving programmed cell death protein 1 (PD-1) or programmed death-ligand 1 (PD-L1) inhibitors. Comprehensive searches were conducted in the Cochrane Library, Web of Science, Embase, and PubMed up to February 13, 2025, adhering to the Preferred Reporting Items for Systematic Reviews and Meta-Analyses (PRISMA) 2020 guidelines. The quality of the included studies was assessed using the Newcastle–Ottawa Scale (NOS), and pooled hazard ratios (HRs) with 95% confidence intervals (CIs) were calculated employing random-effects models. A total of 49 studies encompassing 7,897 patients were analyzed. Elevated NLR was significantly associated with shorter overall survival (OS) (HR = 1.92, 95% CI: 1.70–2.17) and progression-free survival (PFS) (HR = 1.66, 95% CI: 1.48–1.87). These associations remained consistent across most subgroups and sensitivity analyses, including those focused on non-small cell lung cancer (NSCLC) patients with pretreatment NLR measurements. However, it is important to consider heterogeneity and potential publication bias regarding PFS when interpreting these findings. Elevated NLR is indicative of poorer survival outcomes in patients with LC treated with PD-1/PD-L1 inhibitors and may function as a practical prognostic biomarker. Nonetheless, prospective studies with standardized cutoff values are necessary prior to routine clinical implementation.

## Introduction

Lung cancer (LC) is a significant global health burden, accounting for nearly 2.5 million new cases (12.4% of all cancers) and over 1.8 million deaths (18.7% of all cancer-related deaths) in 2022 [1]. It is now the most frequently diagnosed malignancy and a leading cause of cancer mortality worldwide [1]. The prognosis for LC remains poor, with a 5-year survival rate ranging from 10% to 20% [2]. Despite advancements in targeted therapies and chemotherapy that have improved disease control in non-small cell lung cancer (NSCLC), issues such as recurrence and treatment resistance persist [3]. Furthermore, managing small cell lung cancer (SCLC) continues to pose challenges, as chemotherapy remains a crucial treatment option across all stages, yet chemoresistance can develop rapidly [4]. Immune checkpoint inhibitors (ICIs) have recently emerged as a viable treatment option [5]. Notably, the CheckMate 017 trial [6] established nivolumab as the first ICI approved by the U.S. Food and Drug Administration (FDA) for NSCLC, demonstrating significant survival benefits in specific patient populations [7]. The IMpower133 trial has shown the efficacy of atezolizumab, leading to its FDA approval for use in combination with chemotherapy in extensive-stage SCLC [8, 9]. Various programmed cell death protein 1 (PD-1) and programmed death-ligand 1 (PD-L1) inhibitors have been identified to improve survival outcomes among LC patients [10, 11]. However, the response rate to immunotherapy varies widely, making it imperative to identify high-risk LC patients with poor prognoses. Consequently, there is a pressing need to identify reliable prognostic biomarkers.

Inflammation plays a crucial role in the tumor microenvironment, contributing to the initiation, progression, and metastasis of malignant tumors [12, 13]. The neutrophil-to-lymphocyte ratio (NLR)—calculated as the absolute count of neutrophils divided by lymphocytes—serves as a cost-effective marker of systemic inflammation [14]. Numerous studies have indicated that an elevated NLR is associated with unfavorable prognoses across various cancers [15–17]. However, the prognostic utility of NLR in predicting outcomes for LC patients undergoing PD-1 or PD-L1 inhibitor therapy remains unclear. A study by Suh et al. [18] (2018) found that an NLR measured at week 6 serves as a prognostic indicator for NSCLC patients receiving PD-1 inhibitor therapy, with an NLR ≥ 5 correlating with poor overall survival (OS) and progression-free survival (PFS). Conversely, Kutlu et al. [19] found no such correlation in SCLC patients treated with atezolizumab. A recent meta-analysis (MA) assessed the prognostic performance of NLR in advanced solid tumors [20]. This MA included studies measuring NLR prior to treatment and combined various ICIs, such as cytotoxic T-lymphocyte-associated antigen 4 inhibitors. Although subgroup analyses were performed for NSCLC and SCLC, only one study focused on SCLC, and no comprehensive analysis of both LC subtypes was conducted. Consequently, the findings may not adequately reflect the prognostic performance of NLR in broader LC populations.

Given the variability in NLR measurements and its potential prognostic significance, this systematic review and MA aims to evaluate the prognostic value of NLR among individuals with LC receiving PD-1 or PD-L1 inhibitors. The results may provide clinically relevant evidence to inform decision-making.

## Materials and methods

This MA was conducted in accordance with the Preferred Reporting Items for Systematic Reviews and Meta-Analyses 2020 guidelines [21] and was registered with the International Prospective Register of Systematic Reviews (PROSPERO: CRD420251032056).

### Search strategy

The Cochrane Library (OVID), Web of Science, Embase (OVID), and PubMed were searched until February 13, 2025. The search terms included “Neutrophil(s),” “Polymorphonuclear Neutrophils,” “Polymorphonuclear Leukocyte(s),” “LE Cell(s),” “Lymphocyte(s),” “Lymphoid Cell(s),” “Ratio,” “Pulmonary Neoplasm(s),” “Lung Cancer(s),” “Lung Neoplasm(s),” “Pulmonary Cancer(s),” “Cancer of Lung,” “Cancer of the Lung,” “Immune Checkpoint Inhibitor(s),” “Immune Checkpoint Blocker(s),” “Programmed Cell Death Protein 1 Inhibitor(s),” “PD-1,” “Programmed Death Ligand 1 Inhibitors,” “PD-L1,” and “Immunotherapy.” The specific search strategy is detailed in Supplementary Table S1.

### Inclusion and exclusion criteria

The inclusion criteria were as follows: (i) individuals with LC undergoing PD-1 or PD-L1 inhibitors, with or without combination therapies; (ii) studies evaluating the prognostic performance of NLR for predicting OS or PFS; (iii) studies providing hazard ratios (HRs) and 95% confidence intervals (CIs), or sufficient data to compute them; (iv) in cases of overlapping patient populations, only the study with the largest sample size or most comprehensive data was included. Exclusion criteria comprised: (i) reviews, meta-analyses, or case reports; (ii) commentaries, letters to the editor, or conference abstracts; (iii) animal or *in vitro* studies; (iv) studies with incomplete or erroneous data; (v) duplicate publications; (vi) studies lacking accessible full text.

### Data extraction

Two investigators (ZY and GJL) independently reviewed the titles and abstracts of the identified studies. A full-text review was performed to exclude irrelevant studies, with any discrepancies resolved through discussion. Data were extracted independently by the two investigators using Excel 2019. The data collected included the first author, year of publication, study period, region, study type, population, treatment regimen, timing of NLR measurement, sample size, sex distribution, age, tumor-node-metastasis (TNM) stage, NLR cutoff value, HRs with 95% CIs, and outcome measures. To ensure accuracy and independence in data collection, multivariable HRs were prioritized when both univariable and multivariable results were available. For studies reporting HR indirectly, tools such as Engauge Digitizer or WebPlotDigitizer were utilized to extract data from Kaplan-Meier curves. To manage potential data overlap from the same cohort, we identified and flagged overlapping studies by cross-checking authors, affiliated institutions, and patient recruitment periods. In studies with internal validation, only data from the independent validation cohort were included. When NLR was measured at multiple time points (e.g., baseline, post-treatment), only baseline data were used for the primary analysis; data from other time points were included in subgroup analyses examining the timing of measurement effects.

### Quality assessment

Article quality was evaluated using the Newcastle–Ottawa Scale (NOS) [22]. This assessment comprised eight items evaluating selection, comparability, and outcomes, with a maximum score of 9. A score of 7 or above indicated high-quality research. Only studies with a NOS score ≥7 were included in our analysis.

### Statistical analysis

Review Manager (version 5.4) and Stata (version 17) were employed to pool HRs. The I^2^ statistic and Cochran’s *Q* test were utilized to assess heterogeneity across studies, with I^2^ > 50% or *P* < 0.1 indicating significant heterogeneity. A random-effects model (REM) was applied to all analyses, and 95% prediction intervals (PIs) were calculated to account for heterogeneity and to estimate potential effect size ranges in future studies. Subgroup analyses were conducted to identify sources of heterogeneity and assess the robustness of the findings. Sensitivity analyses were performed by sequentially removing each article to evaluate the stability of results. Given the variability in NLR cutoff values and measurement timing, sensitivity analyses were also stratified by these factors. Studies were categorized into three groups based on NLR cutoff values (≤3.43, 3.43–5, ≥5). The value of 3.43 was derived from one included study and falls within commonly used cutoff ranges [19], while 5.0 is a frequently referenced threshold for defining high NLR [23]. This classification aimed to ensure sufficient representation in each subgroup and was exploratory in nature, based on data distribution and statistical power. Additionally, to verify the robustness of core conclusions, sensitivity analyses focused on NSCLC patients with only pretreatment NLR measurements. Publication bias was evaluated using Egger’s test and funnel plots, with *P* < 0.05 indicating statistical significance.

## Results

### Study characteristics

A total of 2,406 studies were initially retrieved. After excluding 843 duplicates, 1,348 studies were removed upon reviewing the titles and abstracts. The complete texts of the remaining articles were subsequently examined. Ultimately, 49 eligible studies [18, 19, 23–69] were included. Figure 1 illustrates the screening process.

**Figure 1.**
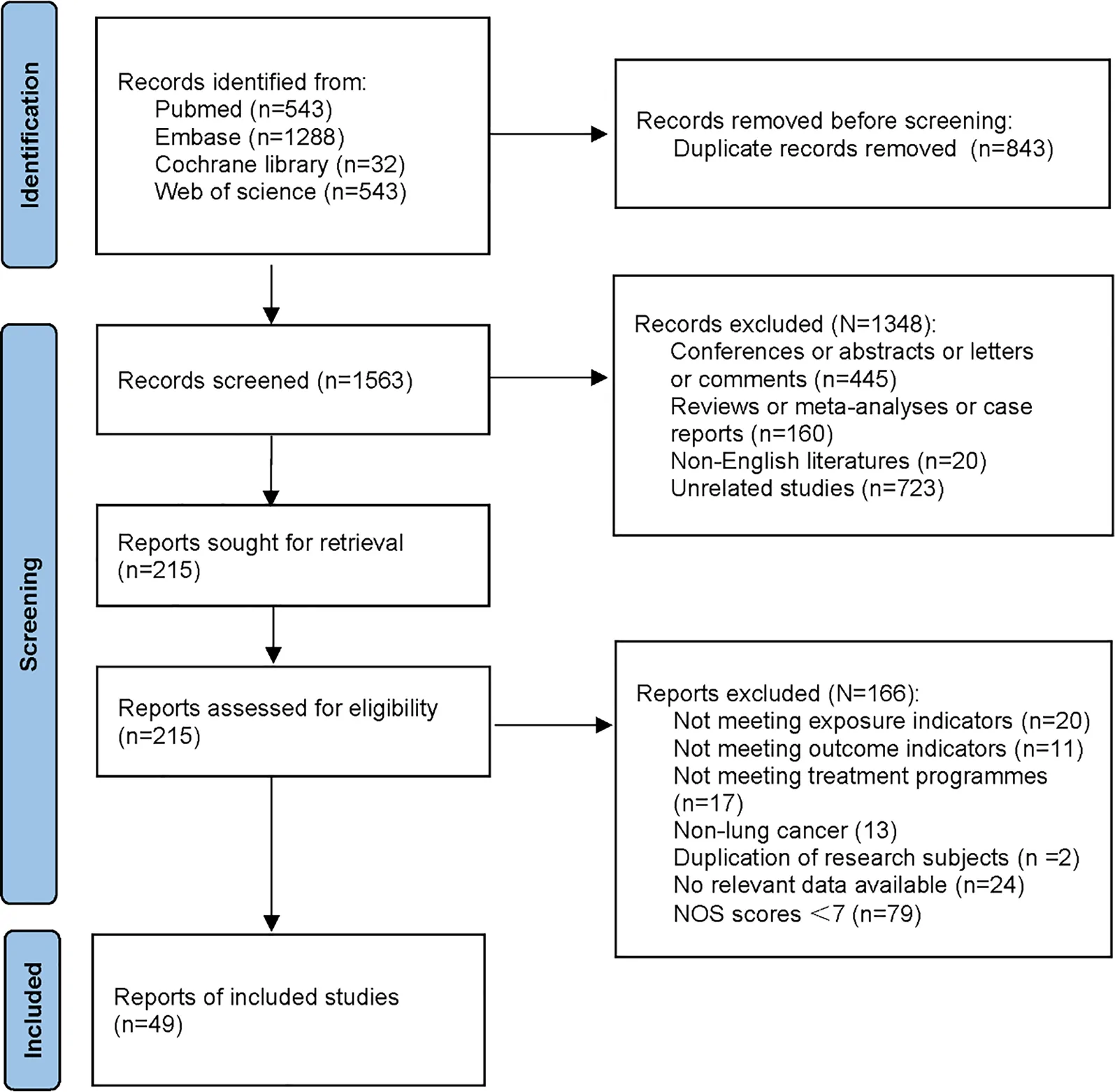
Study selection process.

These 49 studies, published between 2018 and 2025, included 53 comparison groups. Among these, 31 studies were conducted in Asia, 3 in North America, 13 in Europe, and 2 were multinational. Of the studies, 42 focused on NSCLC and 7 on SCLC, encompassing a total of 7,897 patients (5,499 males and 2,398 females). Forty-seven studies reported the mean or median age of the participants, which ranged from 55–76 years. Twenty-six studies employed PD-1 inhibitors, 9 involved PD-L1 inhibitors, and 14 utilized either PD-1 or PD-L1 inhibitors. All 49 studies reported NLR cutoff values, which ranged from 2.1–6.4. The fundamental characteristics of the eligible articles are detailed in Table 1.

**Table 1:** Baseline characteristics of included studies

**Author**	**Study period**	**Region**	**Study type**	**Population**	**Treatment regimen**	**Timing of NLR measurement**	**Sample size (Male/female)**	**Mean/median age**	**TNM stage**	**NLR cut-off**	**Outcome**
Asano 2023 [24]	2016–2019	Japan	R	aNSCLC	PD-1/PD-L1 inhibitors ± chemotherapy	Pre	55 (40/15)	66.3	IIIB/IV	2.1	M:OS
Banna 2020 [25]	2016–2019	Europe	R	aNSCLC	Pembrolizumab	Pre	132 (87/45)	68	I-IV	5	U:OS,PFS
Banna 2021 [26]	2016–2019	Europe	R	aNSCLC	Pembrolizumab	Pre	583 (388/195)	70	NA	4	M:OS
Banna 2022 [27]	2018–2021	UK/ Switzerland	R	aNSCLC	Pembrolizumab + chemotherapy	Pre	308 (171/137)	65	IIIB/IVA/IVB	4	M:OS,PFS
Bolte 2022 [28]	2015–2021	USA	R	LA-NSCLC/mNSCLC	PD-1/PD-L1 inhibitors + other therapy	Pre	92 (48/44)	64	NA	3.24	M:OS
Chen 2021 [29]	2015–2019	China	R	aNSCLC	PD-1 inhibitors ± other therapy	Pre	101 (72/29)	64	IIIB/IV	4.5	M:OS,PFS
Chen 2023 [30]	2021–2022	China	R	aNSCLC	Nivolumab/atezolizumab + chemotherapy	Pre	30 (19/11)	67	III/IV/ recurrence	3.21	M:PFS
Colomb 2024 [31]	2017–2022	France	R	LA-NSCLC	Chemoradiotherapy + durvalumab	Pre-durvalumab	85 (63/22)	65	IIIA-C	2.94	U:OS
Cortellini 2022 [32]	2014–2015	Multi-continents	R	NSCLC	Atezolizumab	Pre	600 (372/228)	63	NA	4	M:OS,PFS
Descourt 2023 [33]	2017–2019	France	R	aNSCLC	Pembrolizumab	Pre	845 (573/272)	65	NA	4	M:OS,PFS
Facchinetti 2018 [34]	2015–2017	Italy	P	LA-NSCLC/ mNSCLC	Nivolumab	Pre	54 (45/9)	69	IIIB/IV	4	M:OS
Fang 2023 [35]	2017–2019	China	R	aNSCLC	PD-1 inhibitors + chemotherapy	Pre	223 (189/34)	60.4	IIIB-C/IV	3.18	M:OS,PFS
He 2021 [36]	2016–2018	China	R	aNSCLC	PD-1/PD-L1 inhibitors	Pre	91 (65/26)	59	NA	3.2	M:OS,PFS
Imai 2021 [37]	2017–2019	Japan	R	aNSCLC	Pembrolizumab	Pre	142 (117/25)	70	III/IV/postoperative recurrence	5	M:OS,PFS
Imai 2023 [38]	2018–2020	Japan	R	Non-squamous NSCLC	Pembrolizumab + chemotherapy	Pre	236 (189/47)	68	II/III/IV/postoperative recurrence	5	M:OS,PFS
Jiang 2024 [39]	2018–2021	China	R	NSCLC	PD-1/PD-L1 inhibitors + other therapy	Pre	148 (126/22)	66	II-III/IV	4.782	M:OS,U:PFS
Kashihara 2023 [40]	2018–2021	Japan	R	LA-NSCLC	chemoradiotherapy + durvalumab	Pre-durvalumab	113 (86/27)	66	IIB/IIIA-C	3.7	U:OS,PFS
Katayama 2020 [41]	2018–2019	Japan	R	aNSCLC	Atezolizumab	Pre	81 (44/37)	71	III/IV/postoperative recurrence	5	M:OS,PFS
Kim 2022 [42]	2017–2020	Korea	R	aNSCLC	Pembrolizumab + chemotherapy	Pre	52 (41/11)	63	NA	6	M:OS,PFS
Krizova 2024 [43]	2018–2022	Czech	P	NSCLC	PD-1/PD-L1 inhibitors ± other therapy	Pre	202 (111/91)	70.1	III/IV	5	U:OS,PFS
Ksienski 2021 [44]	2017–2019	Canada	R	aNSCLC	Pembrolizumab	Pre	220 (99/121)	70	IV/ recurrent nonresectability	6.4	M:OS
Kutlu 2023 [19]	2016–2021	Turkey	R	ES-SCLC	Atezolizumab + chemotherapy	Pre	55 (42/13)	63	Limited/Extensive-stage	3.43	M:OS,PFS
Lu 2022 [45]	2019–2021	China	R	NSCLC	PD-1 inhibitors ± other therapy	Pre	133 (113/20)	58.8	IIIB/IV	3.56	M:OS,PFS
Luo 2024 [46]	2018–2022	China	R	mNSCLC	PD-1 inhibitors + radiotherapy	Pre	117 (100/17)	61	NA	6	M:OS
Luo 2024a [46]	2018–2022	China	R	mNSCLC	PD-1 inhibitors + radiotherapy	Post	117 (100/17)	61	NA	6	M:OS
Matsumoto 2024 [47]	2018–2022	Japan	R	Advanced/recurrent NSCLC	PD-1/PD-L1 inhibitors + other therapy	Pre	280 (223/57)	69	IV or others	5	M:OS,PFS
Nishihara-Kato 2024 [48]	2018–2020	Japan	R	NSCLC	Pembrolizumab + chemotherapy	Pre	144 (121/23)	69	III/IV/post-operative or post-chemoradiotherapy recurrence	5	M:OS,U:PFS
Ohba 2019 [49]	2017–2018	Japan	R	aNSCLC	Pembrolizumab	Pre	32 (29/3)	65	unresectable or postoperative recurrent stage III/ IV	4.11	M:OS
Ozawa 2024 [50]	2018–2023	Japan	R	Advanced/recurrent NSCLC	PD-1/PD-L1 inhibitors + other therapy	Pre	199 (162/37)	NA	III/IV/recurrence	3	M:OS
Pavan 2019 [51]	2013–2018	Italy	R	aNSCLC	PD-1/PD-L1 inhibitors	Pre	184 (125/59)	67.3	NA	3	U:OS,PFS
Prelaj 2019 [52]	2013–2019	Italy	R	aNSCLC	PD-1/PD-L1 inhibitors	Pre	193 (120/73)	65	IIIB-C/IV	4	M:PFS
Qi 2021 [53]	2017–2018	Multi-continents	R	ES-SCLC	Atezolizumab + chemotherapy	Pre	53 (34/19)	NA	Extensive-stage	2.57	M:OS
Riemann 2023 [54]	2020–2021	Germany	P	LA-NSCLC/ mNSCLC	Atezolizumab + chemotherapy	Pre	40 (23/17)	65	NA	6.1	U:OS,PFS
Riemann 2023a [54]	2020–2021	Germany	P	LA-NSCLC/ mNSCLC	Atezolizumab + chemotherapy	Post	40 (23/17)	65	NA	6.1	U:OS
Shiroyama 2018 [55]	2015–2016	Japan	R	aNSCLC	Nivolumab	Pre	201 (135/66)	68	NA	4	U:PFS
Soyano 2018 [56]	2015–2017	USA	R	aNSCLC	PD-1 inhibitors	Pre	157 (83/74)	66	NA	5.9	M:OS,PFS
Stares 2022 [57]	2016–2021	UK	R	aNSCLC	Pembrolizumab	Pre	219 (109/110)	69	NA	5	U:OS,PFS
Stratmann 2022 [58]	2017–2021	Germany	R	Relapsed/refractory SCLC	PD-1/PD-L1 inhibitors ± ipilimumab	Pre	111 (70/41)	63.6	NA	5.8	M:OS
Su 2024 [59]	2019–2022	China	R	aNSCLC	PD-1 inhibitors ± other therapy	Pre	203 (158/45)	64.3	III/IV	6.23	M:OS,PFS
Su 2024a [59]	2019–2022	China	R	aNSCLC	PD-1 inhibitors ± other therapy	Post	203 (158/45)	64.3	III/IV	6.23	U:OS,PFS
Suh 2018 [18]	2013–2016	Korea	R	aNSCLC	PD-1 inhibitors	Post	54 (42/12)	68	NA	5	M:OS,PFS
Takada 2020 [60]	2016–2018	Japan	R	Advanced/recurrent NSCLC	PD-1 inhibitors	Pre	226 (184/42)	66	IIIB/IV/recurrence	6.05	U:OS,PFS
Takeyasu 2021 [61]	2017–2020	Japan	R	Advanced/recurrent NSCLC	Pembrolizumab	Pre	145 (102/43)	67	NA	5	M:PFS
Tian 2025 [62]	2020–2022	China	P	ES-SCLC	PD-L1 inhibitors + chemotherapy	Pre	63 (53/10)	63	Extensive-stage	4.5	M:OS
Ulas 2024 [63]	2019–2024	Turkey	R	aNSCLC	Nivolumab	Pre	104 (94/10)	62	NA	3.03	U:OS,PFS
Wang 2021 [64]	2015–2019	China	R	aNSCLC	PD-1 inhibitors	Pre	105 (73/32)	55	IV	3	M:OS,PFS
Wasamoto 2023 [65]	2019–2021	Japan	R	ES-SCLC	Atezolizumab + chemotherapy	Pre	84 (70/14)	71	III/IV/postoperative recurrence	5	U:OS,PFS
Wasamoto 2025 [66]	2018–2020	Japan	R	Advanced non-squamous NSCLC	Pembrolizumab + chemotherapy	Pre	30 (20/10)	76	III-IV/ postoperative recurrence	5	M:OS,PFS
Xiong 2021 [23]	2015–2018	China	R	SCLC	PD-1/PD-L1 inhibitors ± chemotherapy	Pre	41 (36/5)	61	Limited/extensive stage	5	U:PFS
Xiong 2021a [23]	2015–2018	China	R	SCLC	PD-1/PD-L1 inhibitors ± chemotherapy	Post	41 (36/5)	61	Limited/extensive stage	5	U:PFS
Yuan 2024 [67]	2020–2022	China	R	aNSCLC	PD-1/PD-L1 inhibitors ± other therapy	Pre	107 (84/23)	66	IIIB/IV	3.825	M:OS,PFS
Zattarin 2022 [68]	2019–2021	Italy	P	aNSCLC	Pembrolizumab + chemotherapy	Pre	98 (65/33)	67	IIIB-C/IV	4	M:OS,PFS
Zhang 2024 [69]	2020–2024	China	R	Advanced lung adenocarcinoma	PD-1/PD-L1 inhibitors ± other therapy	Pre	126 (84/42)	65	III/IV	3	M:OS,U:PFS

Entries in the “Author” column that include an “a” suffix (e.g., Luo 2024a) signify post-treatment measurements from the same cohort as their corresponding entries without the suffix (e.g., Luo 2024). In the primary analysis, these paired entries were not considered independent; their post-treatment values were utilized exclusively for subgroup analyses at the assessment timepoint. **Abbreviations:** aNSCLC, advanced non-small cell lung cancer; ES-SCLC, extensive-stage small cell lung cancer; LA-NSCLC, locally advanced non-small cell lung cancer; M, multivariable analysis; mNSCLC, metastatic non-small cell lung cancer; NA, not available; NLR, neutrophil-to-lymphocyte ratio; NSCLC, non-small cell lung cancer; OS, overall survival; P, prospective; PD-1, programmed cell death protein 1; PD-L1, programmed death-ligand 1; PFS, progression-free survival; Post, post-treatment; Pre, pretreatment; R, retrospective; SCLC, small cell lung cancer; TNM, tumor–node–metastasis; U, univariable analysis.

### Quality assessment

As illustrated in Supplementary Table S2, the NOS scores of all 49 articles varied between 7 and 9, indicating that the included studies demonstrated good methodological quality.

### Association between NLR and OS

Forty-four comparison groups, involving 7,287 individuals, were evaluated to examine the association between NLR and OS. Due to significant heterogeneity (I^2^ ═ 53%, *P* < 0.0001), REM was employed. The results revealed that an increased NLR was significantly associated with shorter OS (HR = 1.92, 95% CI: 1.70–2.17, *P* < 0.00001; 95% PI: 1.09–3.38; Figure 2, Table 2). The HR ranged from 0.57–6.67 across the studies.

**Table 2:** Subgroup and sensitivity analyses of the association between NLR and LC prognosis

**Stratification variable**	**Study group**	**HR (95%CI), 95%PI**	***P* value**	**Heterogeneity test**
				***I* ^2^**	***P* value**
**OS**					
**Total**	44	1.92 (1.70–2.17), 1.09–3.38	<0.00001	53%	<0.0001
**Subgroup**					
Region					
North America	3	1.57 (0.89–2.79)	0.12	72%	0.03
Asia	27	1.95 (1.61–2.36)	<0.00001	60%	<0.0001
Europe	12	1.99 (1.67–2.37)	<0.00001	40%	0.07
Multi-continents^a^	2	1.64 (1.35–1.99)	<0.00001	0%	0.90
Study type					
Prospective	5	2.29 (1.54–3.38)	<0.0001	29%	0.23
Retrospective	39	1.89 (1.66–2.15)	<0.00001	56%	<0.0001
Population					
NSCLC	38	1.95 (1.71–2.21)	<0.00001	57%	<0.0001
SCLC	6	1.68 (1.11–2.55)	0.01	25%	0.24
Treatment regimen^b^					
PD-1/PD-L1 inhibitors monotherapy	17	2.22 (1.90–2.59)	<0.00001	47%	0.02
PD-1/PD-L1 inhibitors + other therapy	19	1.54 (1.23–1.94)	0.0002	60%	0.0004
PD-1/PD-L1 inhibitors ± other therapy	8	2.03 (1.65–2.50)	<0.00001	0%	0.81
Treatment regimen^c^					
Atezolizumab	6	1.77 (1.23–2.56)	0.002	44%	0.11
Durvalumab^a^	2	1.10 (0.42–2.86)	0.84	65%	0.09
Nivolumab^a^	2	3.11 (1.46–6.60)	0.003	0%	0.89
Pembrolizumab	13	1.99 (1.59–2.50)	<0.00001	65%	0.0006
Timing of NLR measurement^d,e^					
Post-treatment	4	2.12 (1.02–4.40)	0.04	73%	0.01
Pretreatment	41	1.94 (1.72–2.19)	<0.00001	52%	<0.0001
Pre-durvalumab^a^	2	1.10 (0.42–2.86)	0.84	65%	0.09
Mean/median age					
<65y	14	1.83 (1.47–2.28)	<0.00001	45%	0.04
≥65y	28	1.98 (1.69–2.31)	<0.00001	60%	<0.0001
NLR cut-off					
<3.5	12	1.82 (1.43–2.33)	<0.00001	36%	0.10
≥3.5	32	1.96 (1.70–2.25)	<0.00001	59%	<0.0001
Data source					
Multivariable analysis	34	1.89 (1.65–2.17)	<0.00001	52%	0.0002
Univariate analysis	10	2.02 (1.56–2.62)	<0.00001	55%	0.02
**Sensitivity analysis**					
Pretreatment and NSCLC^f^	35	1.96 (1.73–2.23)	<0.00001	55%	<0.0001
				***I* ^2^**	***P* value**
NLR cut-off					
≤3.43	12	1.82 (1.43–2.33)	<0.00001	36%	0.10
3.43–5.0	13	1.76 (1.41–2.20)	<0.00001	61%	0.002
≥5.0	19	2.10 (1.75–2.51)	<0.00001	53%	0.004
**PFS**					
**Total**	37	1.66 (1.48–1.87), 0.98–2.82	<0.00001	58%	<0.00001
Region					
North America^a^	1	1.61 (1.14–2.27)	0.007	/	/
Asia	26	1.69 (1.41–2.03)	<0.00001	66%	<0.00001
Europe	9	1.67 (1.48–1.89)	<0.00001	3%	0.41
Multi-continents^a^	1	1.34 (1.13–1.59)	0.0008	/	/
Study type					
Prospective	3	1.94 (1.40–2.70)	<0.0001	5%	0.35
Retrospective	34	1.64 (1.45–1.85)	<0.00001	60%	<0.00001
Population					
NSCLC	33	1.67 (1.48–1.88)	<0.00001	61%	<0.00001
SCLC	4	1.57 (0.92–2.66)	0.10	36%	0.19
Treatment regimen^b^					
PD-1/PD-L1 inhibitors monotherapy	16	1.59 (1.36–1.86)	<0.00001	60%	0.001
PD-1/PD-L1 inhibitors + other therapy	14	1.54 (1.29–1.84)	<0.00001	41%	0.06
PD-1/PD-L1 inhibitors ± other therapy	7	2.20 (1.49–3.25)	<0.0001	68%	0.004
Treatment regimen^c^					
Atezolizumab	5	1.64 (1.14–2.36)	0.007	55%	0.06
Durvalumab^a^	1	0.77 (0.44–1.33)	0.35	/	/
Nivolumab^a^	2	1.42 (1.04–1.94)	0.03	0%	0.53
Pembrolizumab	11	1.59 (1.33–1.90)	<0.00001	49%	0.03
Timing of NLR measurement^d,e^					
Post-treatment	3	5.36 (2.46–11.70)	<0.0001	54%	0.11
Pretreatment	35	1.65 (1.49–1.83)	<0.00001	48%	0.0009
Pre-durvalumab^a^	1	0.77 (0.44–1.33)	0.35	/	/
Mean/median age					
<65y	11	1.76 (1.29–2.42)	0.0004	72%	0.0001
≥65y	26	1.63 (1.45–1.84)	<0.00001	50%	0.002
NLR cut-off					
<3.5	8	1.43 (1.19–1.72)	0.0002	0%	0.61
≥3.5	29	1.73 (1.51–1.98)	<0.00001	65%	<0.00001
Data source					
Multivariable analysis	23	1.76 (1.49–2.07)	<0.00001	67%	<0.00001
				***I* ^2^**	***P* value**
Univariate analysis	14	1.59 (1.37–1.84)	<0.00001	33%	0.11
**Sensitivity analysis**					
Pretreatment and NSCLC^f^	31	1.66 (1.49–1.85)	<0.00001	51%	0.0007
NLR cut-off					
≤3.43	8	1.43 (1.19–1.72)	0.0002	0%	0.61
3.43–5.0	11	1.49 (1.30–1.72)	<0.00001	38%	0.10
≥5.0	18	1.92 (1.57–2.36)	<0.00001	69%	<0.00001

a, Number of studies included: ≤2; b, Stratified by combination therapy with PD-1/PD-L1 inhibitors; c, Stratified by PD-1/PD-L1 inhibitors type; d, Combined subgroup and sensitivity analysis; e, The subgroup analysis of assessment time included repeated measurements from the same cohort (e.g., pre- and post-treatment NLR). These paired data were analyzed as separate entries only within this subgroup to ensure that each cohort was included only once in the main pooled analysis; f, NSCLC patients with only pre-treatment NLR measurements. **Abbreviations:** CI, confidence interval; HR, hazard ratio; I^2^, inconsistency statistic; LC, lung cancer; NLR, neutrophil-to-lymphocyte ratio; NSCLC, non-small cell lung cancer; OS, overall survival; PD-1, programmed cell death protein 1; PD-L1, programmed death-ligand 1; PFS, progression-free survival; PI, prediction interval; SCLC, small cell lung cancer.

**Figure 2.**
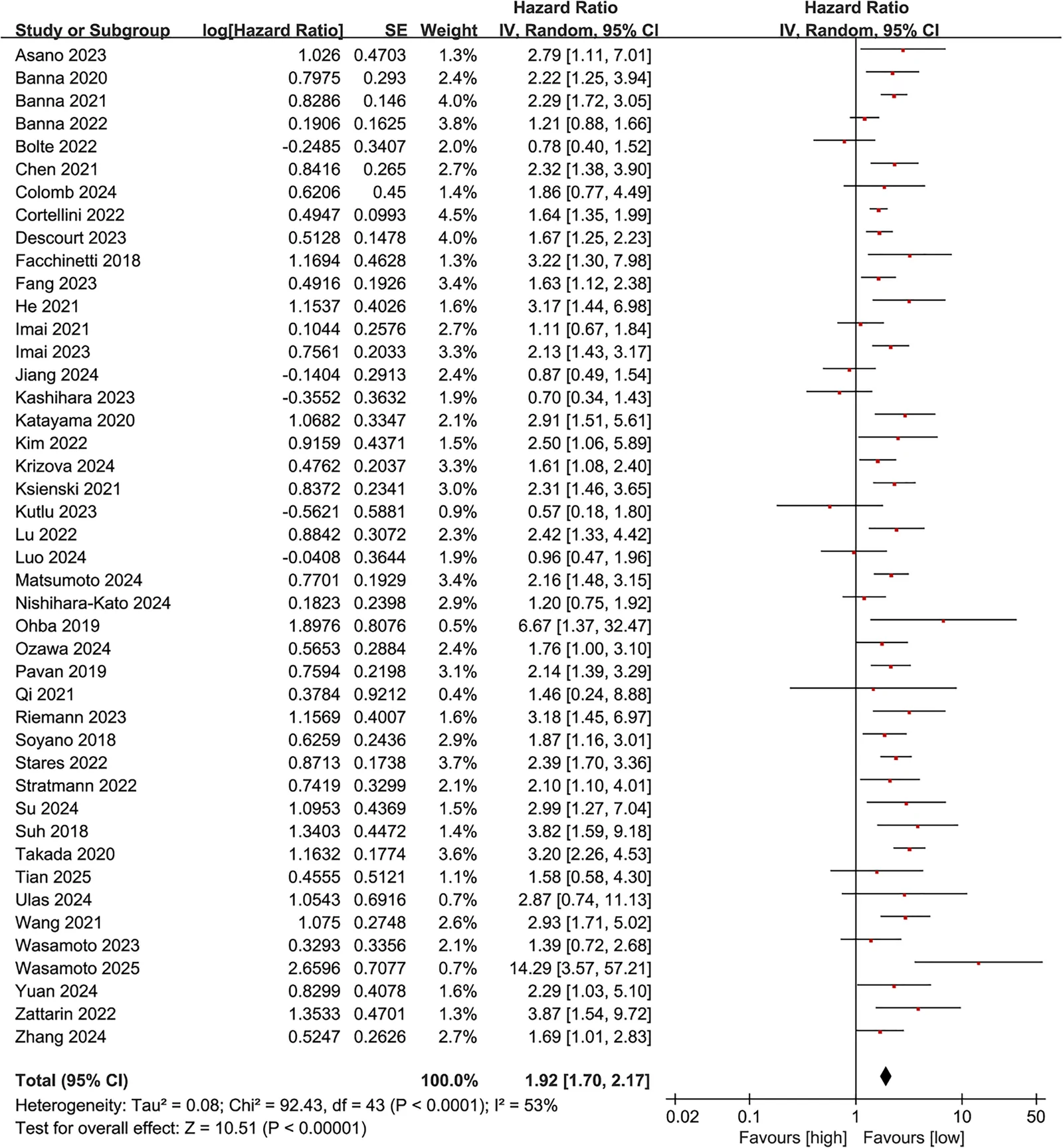
**Forest plot of the association between NLR and OS.** The forest plot summarizes 44 comparison groups including 7,287 patients with LC treated with PD-1/PD-L1 inhibitors. Because significant between-study heterogeneity was observed (I^2^ ═ 53%, *P* < 0.0001), pooled estimates were calculated using a REM. Elevated NLR was significantly associated with shorter OS compared with low NLR (HR = 1.92, 95% CI: 1.70–2.17, *P* < 0.00001; 95% PI: 1.09–3.38), supporting its prognostic relevance in this patient population. **Abbreviations:** CI, confidence interval; HR, hazard ratio; I^2^, inconsistency statistic; LC, lung cancer; NLR, neutrophil-to-lymphocyte ratio; OS, overall survival; PD-1, programmed cell death protein 1; PD-L1, programmed death-ligand 1; PI, prediction interval; REM, random-effects model.

### Subgroup analysis of OS

Subgroup analyses stratified by study type, population, treatment regimen, geographic region, timing of NLR measurement, average or median age, NLR cutoff, and HR data source were performed. The data indicated no statistically significant association between NLR and OS in the North American subgroup (HR = 1.57, 95% CI: 0.89–2.79, *P* ═ 0.12), the durvalumab treatment subgroup (HR = 1.10, 95% CI: 0.42–2.86, *P* ═ 0.84), and the pre-durvalumab subgroup (HR = 1.10, 95% CI: 0.42–2.86, *P* ═ 0.84). However, the wide confidence intervals suggest uncertainty in these findings, preventing definitive conclusions. In contrast, NLR exhibited a significant association with OS in other subgroups. The subgroup analyses indicated that region, study type, population, treatment regimen, and NLR cutoff value may be potential sources of heterogeneity (Table 2). Given the limited number of studies in some subgroups, these results should be considered preliminary and require validation in future research.

### Association between NLR and PFS

Thirty-seven comparison groups, comprising 6,233 individuals, were analyzed to investigate the association between NLR and PFS. A REM was applied due to significant heterogeneity (I^2^ ═ 58%, *P* < 0.00001). The findings suggested that individuals with elevated NLR exhibited significantly shorter PFS compared to those with low NLR (HR = 1.66, 95% CI: 1.48–1.87, *P* < 0.00001; 95% PI: 0.98–2.82; Figure 3; Table 2). The HR ranged from 0.77–5.11 across the studies.

**Figure 3.**
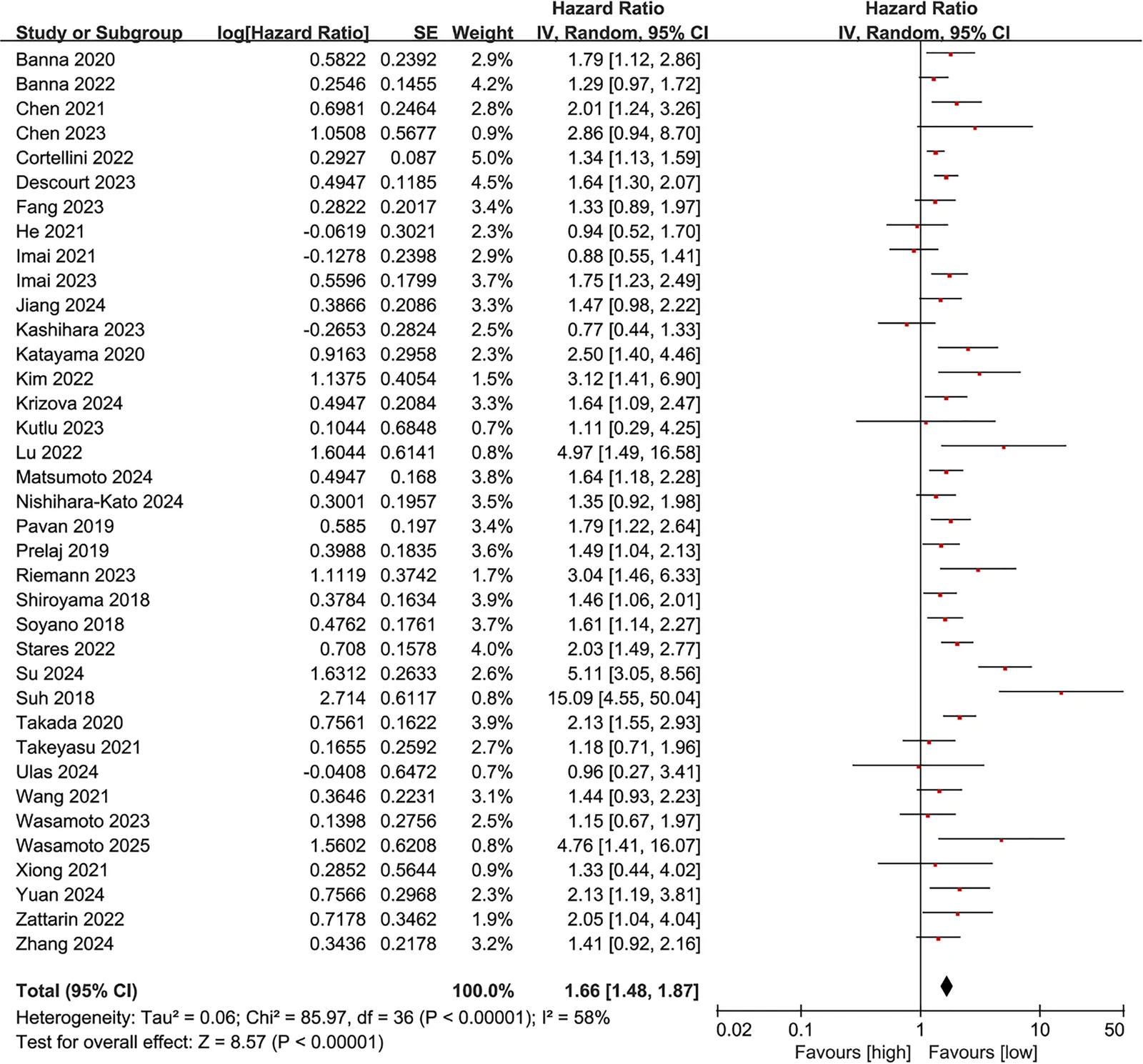
**Forest plot of the association between NLR and PFS.** The forest plot summarizes 37 comparison groups including 6,233 patients with LC receiving PD-1/PD-L1 inhibitors. Because significant between-study heterogeneity was observed (I^2^ ═ 58%, *P* < 0.00001), pooled estimates were calculated using a REM. Elevated NLR was significantly associated with shorter PFS compared with low NLR (HR = 1.66, 95% CI: 1.48–1.87, *P* < 0.00001; 95% PI: 0.98–2.82), although the PI indicates that the magnitude of this association may vary across future clinical settings. **Abbreviations:** CI, confidence interval; HR, hazard ratio; I^2^, inconsistency statistic; IV, inverse variance; LC, lung cancer; NLR, neutrophil-to-lymphocyte ratio; PD-1, programmed cell death protein 1; PD-L1, programmed death-ligand 1; PFS, progression-free survival; PI, prediction interval; REM, random-effects model; SE, standard error.

### Subgroup analysis of PFS

Subgroup analyses stratified by population, region, timing of NLR measurement, study type, mean or median age, treatment regimen, NLR cutoff values, and HR data source were conducted. The results indicated no statistically significant association between NLR and PFS in the SCLC subgroup (HR = 1.57, 95% CI: 0.92–2.66, *P* ═ 0.10), the durvalumab treatment subgroup (HR = 0.77, 95% CI: 0.44–1.33, *P* ═ 0.35), and the pre-durvalumab subgroup (HR = 0.77, 95% CI: 0.44–1.33, *P* ═ 0.35). Conversely, NLR demonstrated significant prognostic value for PFS in all other subgroups. The subgroup analyses suggested that region, study type, population, treatment regimen, timing of NLR measurement, NLR cutoff value, and HR data source may be potential sources of heterogeneity. Table 2 provides the results of the subgroup analyses. Due to the limited number of studies included in some subgroups, these results should be interpreted with caution and validated in future studies.

### Sensitivity analysis

Sensitivity analyses were conducted to assess the robustness of the association between NLR and OS or PFS. After sequentially removing individual studies, the effect sizes remained consistent, indicating that no single article significantly influenced the pooled estimates for OS (Figure 4) or PFS (Figure 5), suggesting that the primary findings are reasonably robust. Stratified analysis by NLR cutoff values (≤3.43, 3.43–5, ≥5) revealed that the direction of the combined effect across all cohorts was consistent with the main analysis (Table 2). Additionally, stratified analysis by the timing of NLR measurement demonstrated that both pretreatment and post-treatment high NLR were associated with shorter OS and PFS, aligning with the main analysis. Notably, no statistically significant association of NLR with OS and PFS was observed in the pre-durvalumab treatment cohort (Table 2). Due to the limited number of cohort studies, these results should be interpreted with caution.

**Figure 4.**
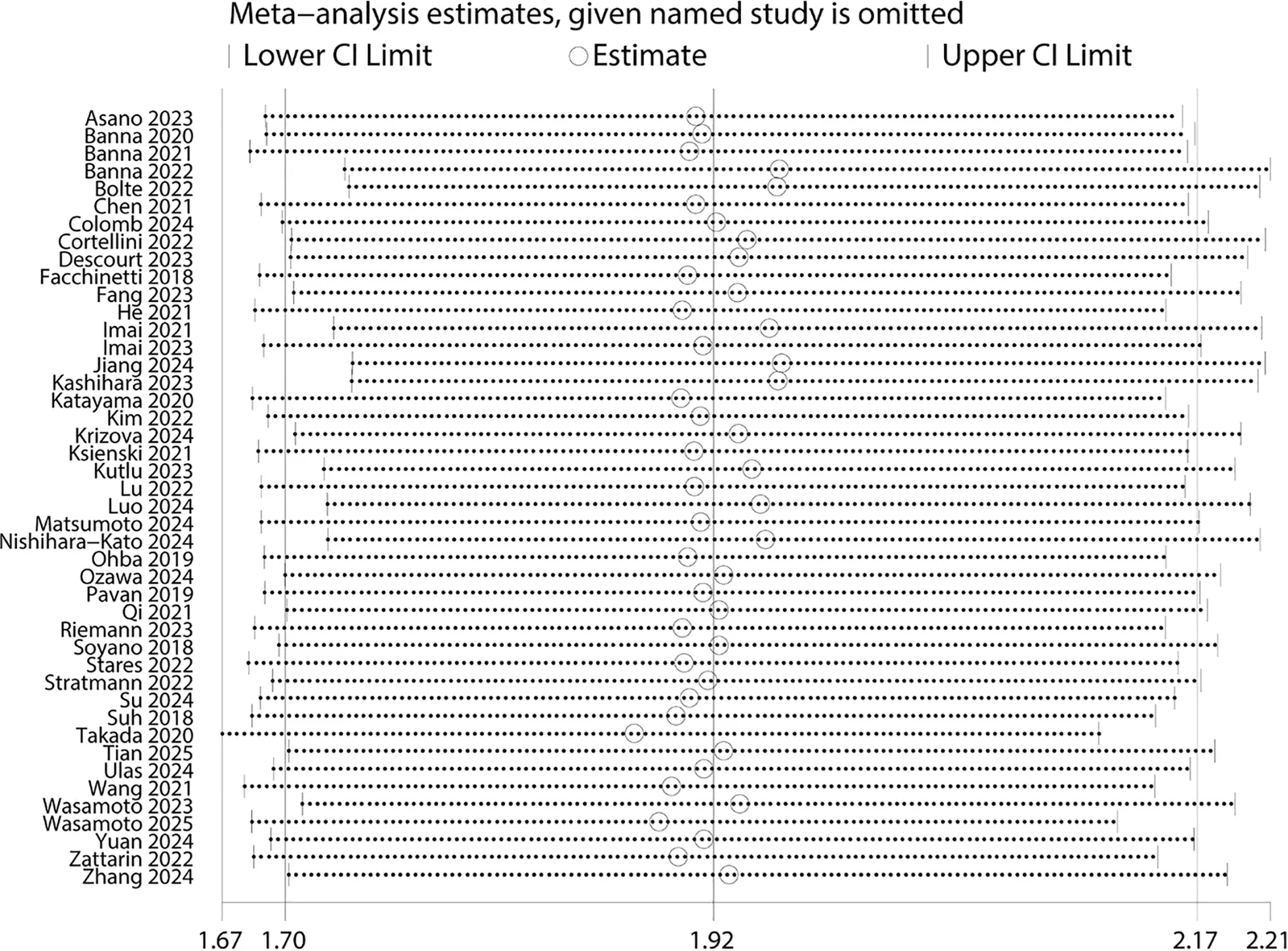
**Leave-one-out sensitivity analysis of the association between NLR and OS.** Sensitivity analysis was performed by sequentially omitting each study from the meta-analysis and recalculating the pooled HR for OS. The pooled estimates and corresponding 95% CIs remained stable after exclusion of any individual study, indicating that no single study substantially influenced the overall association between elevated NLR and shorter OS. **Abbreviations:** CI, confidence interval; HR, hazard ratio; NLR, neutrophil-to-lymphocyte ratio; OS, overall survival.

**Figure 5.**
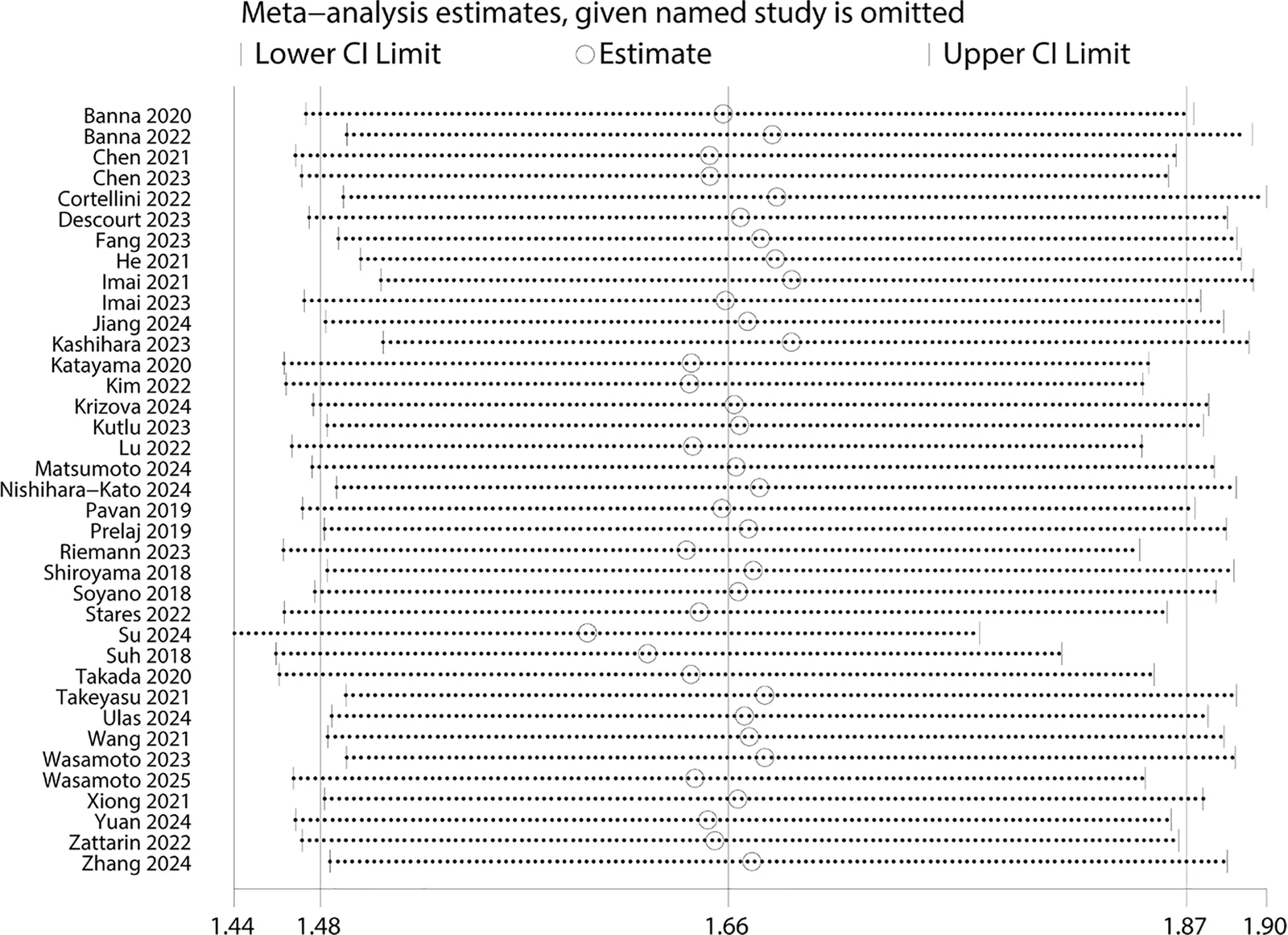
**Leave-one-out sensitivity analysis of the association between NLR and PFS.** Sensitivity analysis was performed by sequentially omitting each study from the meta-analysis and recalculating the pooled HR for PFS. The pooled estimates and corresponding 95% CIs remained stable after exclusion of any individual study, indicating that no single study substantially influenced the overall association between elevated NLR and shorter PFS. **Abbreviations:** CI, confidence interval; HR, hazard ratio; NLR, neutrophil-to-lymphocyte ratio; PFS, progression-free survival.

Furthermore, a sensitivity analysis was conducted on NSCLC patients for whom NLR was measured prior to treatment. The results indicated that a high NLR was associated with shorter OS (HR = 1.96, 95%CI: 1.73–2.23, *P* < 0.00001, I^2^ ═ 55%) and PFS (HR = 1.66, 95%CI: 1.49–1.85, *P* < 0.00001, I^2^ ═ 51%); however, heterogeneity did not significantly decrease (Table 2).

### Publication bias

The funnel plot for OS displayed general symmetry (Figure 6), and the Egger’s test indicated no publication bias (*P* ═ 0.228). Although the funnel plot for PFS appeared roughly symmetrical (Figure 7), the results of the Egger’s test suggested the presence of publication bias (*P* ═ 0.042). The trim-and-fill method was employed to evaluate the impact of publication bias on the results. The findings indicated that funnel plot symmetry could be achieved without interpolating any studies; therefore, the pooled HR remained unchanged (HR = 1.66, 95% CI: 1.48–1.87). Given that the Egger’s test suggested the presence of publication bias, the pooled estimates for NLR and PFS should be interpreted with caution.

**Figure 6.**
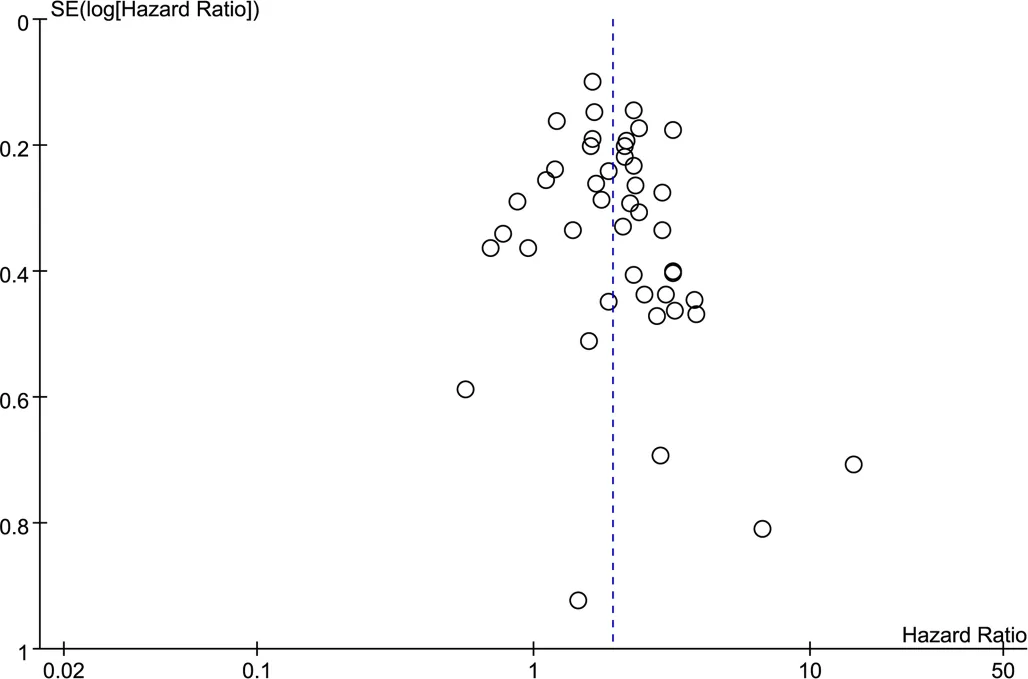
**Funnel plot assessing publication bias for the association between NLR and OS.** The funnel plot displays the distribution of study-specific HRs against their SEs for the association between NLR and OS. Visual inspection showed an approximately symmetrical distribution around the pooled effect estimate, suggesting no evident publication bias or small-study effect. This finding was supported by Egger’s test, which did not indicate significant publication bias (*P* ═ 0.228). **Abbreviations:** HR, hazard ratio; NLR, neutrophil-to-lymphocyte ratio; OS, overall survival; SE, standard error.

**Figure 7.**
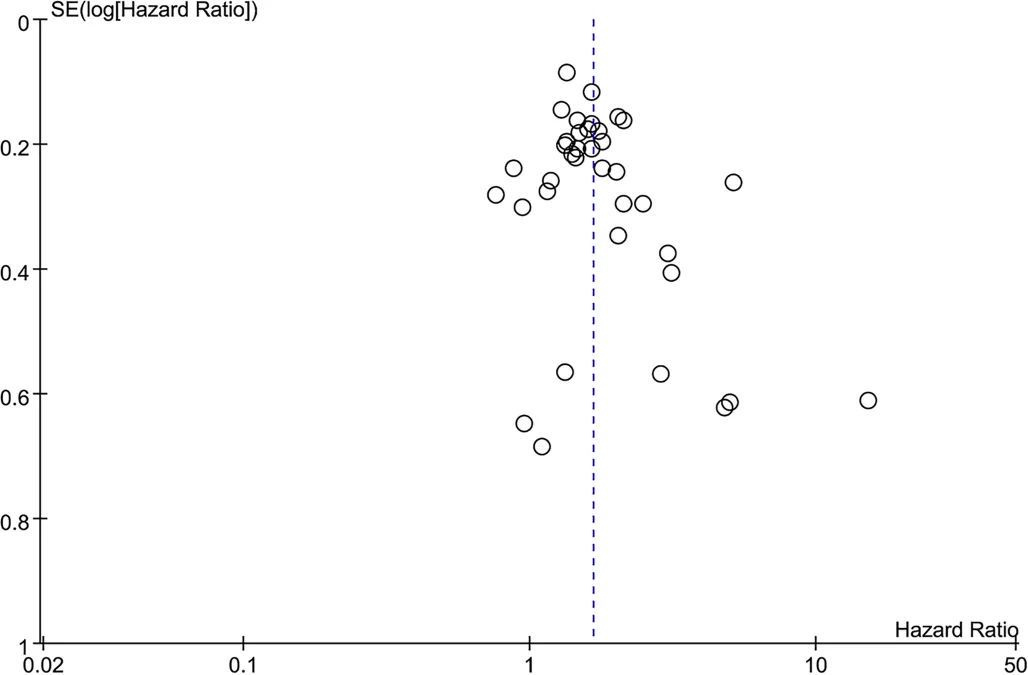
**Funnel plot assessing publication bias for the association between NLR and PFS.** The funnel plot displays study-specific HRs plotted against their SEs for the association between NLR and PFS. Although visual inspection suggested an approximately symmetrical distribution around the pooled effect estimate, Egger’s test indicated potential publication bias or small-study effects (*P* ═ 0.042). Therefore, the pooled estimate for PFS should be interpreted with caution. **Abbreviations:** HR, hazard ratio; NLR, neutrophil-to-lymphocyte ratio; PFS, progression-free survival; SE, standard error.

## Discussion

Systemic inflammation is a hallmark of malignancy and correlates with clinical outcomes in cancer patients [70]. The NLR, a readily available and routinely monitored marker of inflammation, has garnered increasing attention for its prognostic value in LC. Suzuki et al. observed that a higher NLR before treatment is associated with unfavorable OS in individuals with limited SCLC [71]. Similarly, Huang et al. found that elevated NLR correlates with inferior OS and PFS among NSCLC patients undergoing chemotherapy [72]. However, the prognostic significance of NLR across the entire LC population, including both NSCLC and SCLC patients receiving PD-1 or PD-L1 inhibitor therapy, remains unclear.

This study synthesized evidence from 49 studies involving 7,897 individuals with LC undergoing PD-1 or PD-L1 inhibitor therapy. The data demonstrated a marked association between elevated NLR and worse PFS and OS. Sensitivity analyses revealed that excluding any individual study did not significantly alter the direction or significance of the association, indicating that the primary findings are robust to a certain extent. Furthermore, the direction of combined effects for NSCLC patients with pretreatment NLR measurements and cohorts stratified by NLR cutoff values was consistent with the main analysis. According to stratified analysis by the timing of NLR measurement, results for both pretreatment and post-treatment time points were consistent with the main analysis. Notably, no statistically significant association of NLR with OS and PFS was observed in the pre-durvalumab cohort, which may be attributed to the limited number of studies included. The Egger’s test suggested significant publication bias in PFS. Although adjusted analyses continued to support a consistent association between NLR and poor prognosis, the estimated effect size should still be interpreted with caution.

Previous meta-analyses have examined the prognostic value of NLR among LC patients undergoing immunotherapy. Su et al. (2025) indicated that a high NLR prior to treatment is significantly correlated with poorer OS and PFS in advanced NSCLC [20]. Similarly, Nguyen et al. highlighted the prognostic utility of NLR in NSCLC within a MA [73], although their literature search was confined to studies published until October 2021. Other studies have yielded similar findings [74–76]. However, these prior analyses involve fewer studies, earlier data, and primarily focus on NSCLC patients.

In comparison to previous studies [20, 73–76], this MA possesses several notable strengths. Firstly, a more rigorous selection approach was employed, utilizing the NOS and including only studies with scores of seven or higher. This strategy reduces potential confounding factors and enhances the reliability of the results. Secondly, our study population is larger, integrating SCLC with NSCLC for the first time, thereby offering a comprehensive evaluation of LC and addressing the research gap concerning SCLC. Thirdly, the data included are more current, encompassing 49 publications and 7,897 patients, making this the most extensive and up-to-date analysis in this field, yielding more timely and robust conclusions. Finally, our study conducted in-depth subgroup analyses to identify populations in which NLR has the greatest prognostic relevance and the conditions under which it may be most applicable. A novel subgroup analysis stratified by individual PD-1/PD-L1 inhibitors was also performed to investigate whether the prognostic utility of NLR is moderated by the specific type of ICI administered.

Subgroup analyses were conducted based on study type, patient population, region, treatment strategy, the timing of NLR measurement, mean or median age, NLR cutoff values, and the source of HR data. The results indicated no statistically significant association between NLR and OS or PFS in either the durvalumab treatment subgroup or the pre-durvalumab subgroup. Furthermore, no significant association between NLR and OS was observed in the North American subgroup, nor between NLR and PFS in the SCLC subgroup, potentially due to limited sample sizes in these cohorts. Therefore, large-scale, multicenter, international studies are necessary to verify regional and racial differences in the prognostic utility of NLR. Additionally, durvalumab is typically used as consolidation therapy following concurrent chemoradiotherapy [77]. However, chemoradiotherapy may alter peripheral blood cell counts, potentially diminishing the predictive power of NLR. In the remaining subgroups, NLR was significantly associated with both OS and PFS.

To explore the sources of heterogeneity, we performed an extensive subgroup analysis. The results indicated that heterogeneity within each subgroup did not consistently decrease compared to the main analysis, suggesting that region, study type, population, treatment regimen, NLR cutoff, timing of NLR measurement, and source of HR data may be potential sources of variability. These factors could collectively contribute to the discrepancies observed across studies. Given the limited number of studies included in certain subgroups, the observed differences may reflect confounding factors such as study design and population characteristics rather than true biological effects.

This study combined analyses of NSCLC and SCLC, encompassing different disease stages, immunotherapy regimens, and NLR values measured before and after treatment. However, these scenarios are not entirely equivalent, as NSCLC and SCLC differ in pathophysiology, biological behavior, treatment protocols, and prognosis [78]. The extent of the inflammatory response may vary based on pathological type and disease stage [79], and different immunotherapy regimens may also exert varying effects on patient prognosis [80]. These factors may serve as important sources of clinical heterogeneity. Future research should stratify baseline treatment strategies to more accurately assess the independent prognostic value of NLR in specific treatment contexts.

NLR, as a prognostic biomarker in various diseases, reflects the balance between inflammation and immunity [81]. The mechanisms linking NLR to poor prognosis in cancer patients remain to be fully elucidated, although several hypotheses have been proposed. Neutrophils can promote tumor initiation and progression through multiple pathways. Existing evidence suggests that neutrophils facilitate tumor colonization by producing reactive oxygen species and activating IL-1β secretion [82]. Angiogenesis significantly contributes to cancer progression, and neutrophils enhance this process by secreting factors such as prokineticin 2 (Bv8), matrix metalloproteinase 9, and vascular endothelial growth factor [83]. Additionally, neutrophils can awaken dormant tumor cells, capture circulating tumor cells, and facilitate tumor recurrence and metastasis by releasing neutrophil extracellular traps [84]. Moreover, neutrophils exhibit immunosuppressive effects; tumor-associated neutrophils can promote tumor growth by inducing apoptosis of CD8^+^ T cells via tumor necrosis factor alpha (TNF-α) and nitric oxide-dependent mechanisms [85].

Current evidence demonstrates that, in NSCLC, elevated neutrophil counts correlate with decreased CD8+ T cell infiltration [86]. Arasanz et al. reported that low-density neutrophils may mediate primary resistance to ICIs by activating the hepatocyte growth factor/cellular mesenchymal–epithelial transition factor (c-MET) signaling pathway [87]. Lymphocytes are considered key effector cells in antitumor immunity [74]. Reduced lymphocyte counts indicate impaired cell-mediated immune responses [88], and increased lymphocyte infiltration correlates with improved clinical outcomes in solid tumors [89]. Therefore, among LC patients receiving PD-1 or PD-L1 inhibitor therapy, a high NLR may signify a poor prognosis, while a low NLR may indicate favorable clinical outcomes. These findings suggest that NLR may serve as a promising prognostic biomarker. Nonetheless, its application in clinical settings requires validation through prospective studies. Future research should establish standardized cutoff values for NLR and assess its incremental value relative to existing clinical models, as this study does not provide such evidence.

Several limitations should be acknowledged. First, heterogeneity was present across the included studies. Although subgroup analyses were conducted to explore the sources of heterogeneity, they did not fully elucidate it. Therefore, the pooled HR should be interpreted as a general trend rather than a precise estimate. Additionally, the relatively wide 95% PI indicates that the true effect may vary across different clinical settings, a consideration essential for interpreting the results. Second, all included studies were published in English, and many adopted retrospective designs. Due to the limited number of articles in certain subgroup analyses, publication bias may exist. Third, as the study population predominantly comprised individuals from Asia, caution is warranted when generalizing these results to other regions. Moreover, given the limited data on SCLC in the included studies, future large-scale research is needed to validate these findings. Fourth, treatment regimens (e.g., drug type, monotherapy vs. combination therapy) varied across studies. Furthermore, since this analysis relied solely on exploratory comparisons of study-level subgroups and did not account for individual baseline characteristics or concomitant treatments (e.g., chemotherapy or radiation therapy), the differences in results should be interpreted with caution as hypothesis-generating. Fifth, the inclusion of LC patients at varying stages of treatment and disease progression, as well as differences in the timing of NLR measurements and cutoff values, may contribute to heterogeneity across studies, thereby affecting the stability of the pooled effect size. Sixth, the analysis of post-treatment NLR may be subject to immortal time bias, as it included only patients who survived long enough to undergo post-treatment blood testing. Seventh, due to significant variations in adjustments for age, disease stage, and physical condition across different studies, as well as incomplete reporting, a rigorous sensitivity analysis could not be conducted, leaving residual risk of bias. Finally, a high NOS score does not fully mitigate the methodological issues inherent in studies of prognostic factors, which may lead to potential selection bias.

## Conclusion

This study demonstrated that an elevated NLR is significantly associated with poorer OS and PFS in LC patients receiving PD-1/PD-L1 inhibitors, suggesting that NLR may serve as a promising prognostic biomarker. Future large-scale prospective multicenter research employing standardized methodologies is necessary to validate the performance of NLR in assessing prognosis.

## Supplemental data

Supplemental data are available at the following link: https://www.bjbms.org/ojs/index.php/bjbms/article/view/14145/4207.

## Data Availability

The data that support the findings of this study are available from the corresponding author upon reasonable request.
